# Changing Battlelines: 12-Month Antimicrobial Resistance Trends at a Tertiary Care Hospital in Nagpur, Maharashtra, India

**DOI:** 10.7759/cureus.100797

**Published:** 2026-01-05

**Authors:** Smita V Mohod, Arya Rajan, Devi Asokan, Pritam Das, Gopal Agrawal

**Affiliations:** 1 Microbiology, Indira Gandhi Government Medical College and Hospital, Nagpur, IND

**Keywords:** antibiotic sensitivity test, antimicrobial resistance, disc diffusion test, methicillin-resistant staphylococcus aureus, vancomycin-resistant enterococcus

## Abstract

Background: The global surge in antimicrobial resistance (AMR) presents a significant public health threat, especially in developing nations like India with high infectious disease burdens and unregulated antibiotic use. Surveillance of bacterial pathogens and their resistance profiles is essential to inform empirical treatment and formulate antibiotic stewardship policies. This study was conducted to evaluate the bacteriological profile and antimicrobial resistance patterns of clinical isolates over a 12-month period at a tertiary care hospital. The findings aim to guide empirical antibiotic therapy and strengthen hospital antibiotic stewardship policies through the development of a local antibiogram.

Methods: A retrospective, observational study was conducted in the Microbiology Laboratory of Indira Gandhi Government Medical College and Hospital in Nagpur, Maharashtra, India, from May 2024 to April 2025. Out of a total of 22,125 clinical specimens, 4603 (22.87%) were bacterial culture positive, and identification was done using standard conventional microbiological procedures. Antimicrobial susceptibility testing (AST) was performed in accordance with the Clinical and Laboratory Standards Institute (CLSI) guidelines M100 and M45 for *Salmonella typhi* and *Vibrio cholerae*. Data was analyzed.

Results: Of the total samples, 4603 (22.87%) yielded positive cultures. Gram-negative organisms predominated (80.42%). Among these, *Escherichia coli* (35.7%), *Klebsiella pneumoniae* (28.4%), *Pseudomonas aeruginosa* (21.1%), and *Acinetobacter* spp. (13%) were the most common pathogens. Of the 901 (19.6%) Gram-positive isolates, 73.7% were *Staphylococcus aureus*, of which 66.1% were methicillin-resistant *Staphylococcus aureus* (MRSA).* Enterococcus* comprised 152 (16.9%) isolates, and out of these, 4.6% isolates were vancomycin-resistant* Enterococcus* (VRE). High resistance rates were observed among Gram-negative organisms to third-generation cephalosporins (>70%) and fluoroquinolones (>40%). Among Gram-positive bacteria, resistance to penicillin was nearly universal (>95%), while linezolid resistance remained low (8.1%).

Conclusion: The study reveals a high prevalence of multidrug-resistant organisms in clinical samples, emphasizing the urgent need for routine surveillance, infection control measures, and rational antibiotic usage. Data generated from such studies can guide empirical therapy and help to develop regional antibiograms.

## Introduction

Bacterial antimicrobial resistance (AMR), which occurs when changes in bacteria cause the drugs used to treat infections to be less effective, has emerged as one of the leading public health threats of the 21st century [1]. The misuse and overuse of antibiotics have led to the adaptation of microorganisms, resulting in AMR [2]. To address this, the World Health Organization (WHO) promotes initiatives such as the World Antimicrobial Awareness Week, while India follows the Indian Council of Medical Research (ICMR) guidelines that regulate empirical antibiotic use, encourage narrow-spectrum "Access" antibiotics, and monitor antibiotic combinations. AMR occurs when microorganisms like bacteria, viruses, fungi, and parasites resist antimicrobial drugs, which also increases the risk of infection [3]. The ESKAPE (*Enterococcus faecium*, *Staphylococcus aureus*, *Klebsiella pneumoniae*, *Acinetobacter baumannii*, *Pseudomonas aeruginosa*, and *Enterobacter *species) pathogens are common causes of life-threatening nosocomial infections among critically ill and immunocompromised individuals and are characterized by potential drug resistance mechanisms [4]. AMR is a leading cause of death around the world, with the highest burdens in low-resource settings [1]. AMR is a major global threat affecting human health. If no action is taken, it will cause 10 million deaths annually by 2050 [5]. Moreover, to tackle this alarming upsurge in AMR incidence, the WHO has prompted healthcare providers to implement antimicrobial stewardship (AMS) programs to restrain the inappropriate use of antimicrobials [6]. Hospital-based surveillance of microbial pathogens and their resistance profiles is critical for guiding empirical therapy, optimizing infection control policies, and curbing the development of multidrug-resistant (MDR) organisms. Therefore, this study aims to characterize the prevailing resistance trends of key clinically significant culture-positive pathogens in a tertiary care teaching hospital.

## Materials and methods

A retrospective, observational study was conducted in the Microbiology Laboratory of Indira Gandhi Government Medical College and Hospital in Nagpur, Maharashtra, India, from May 2024 to April 2025, involving patients attending the outpatient and inpatient departments of the hospital. During this time, a total of 22,125 clinical specimens were received for microbiological evaluation.

All non-duplicate bacterial isolates from patient specimens that were adequately collected, properly labelled, and processed according to microbiological protocols were included in the study. A wide spectrum of clinical samples was analyzed, including blood, urine, pus/wound swabs, stool, cervico-vaginal swab, body fluids including cerebrospinal fluid and pleural fluid, and respiratory specimens including sputum, bronchoalveolar lavage (BAL), and endotracheal aspirates.

Specimens were processed in accordance with standard microbiological techniques [7]. Initial evaluation involved Gram staining or wet mount and culture on appropriate media depending upon the type of sample and clinical history, followed by identification based on colony characteristics, microscopy, and conventional biochemical reactions. Wherever required, additional confirmatory conventional tests were applied for accurate species-level identification.

Antimicrobial susceptibility was determined by conventional methods like the Kirby-Bauer disc diffusion method, E-strip method, and broth microdilution method as per recommended by the Clinical and Laboratory Standards Institute (CLSI) guidelines M100 [8] and M45 [9] for *Salmonella typhi* and *Vibrio cholerae*.

Operational definitions

Methicillin-resistant *Staphylococcus aureus* (MRSA) was defined by using cefoxitin as a surrogate marker in disc diffusion testing as it indicates the presence of the chromosomally coded mec A gene [10].

Vancomycin-resistant *Enterococcus *(VRE) was identified based on disc diffusion test with vancomycin and teicoplanin consistent with acquired resistance determinants such as van A or van B [10]. The vancomycin resistance was confirmed by the broth dilution method.

MDR organisms were classified as those exhibiting non-susceptibility to at least one antimicrobial agent in ≥3 antimicrobial classes tested [10].

All laboratory data were entered into Microsoft Excel (Microsoft Corporation, Redmond, Washington, United States) and subsequently analyzed accordingly. The prevalence of bacterial isolates, their resistance patterns, and the distribution of multidrug resistance were expressed as percentages. Comparative analysis between Gram-positive and Gram-negative organisms was also carried out. Quality control of antimicrobial susceptibility testing (AST) was ensured by testing reference strains (*Escherichia coli* ATCC 25922, *Staphylococcus aureus* ATCC 25923, *Pseudomonas aeruginosa* ATCC 27853, *Enterococcus faecalis* ATCC 29212, and *Enterococcus faecium* ATCC 35667) in parallel with clinical isolates.

## Results

**Figure 1:**
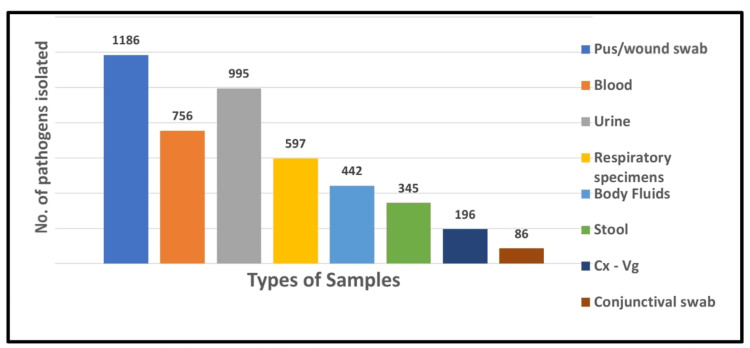
Sample-wise distribution of culture-positive isolates (n=4603) Cx-Vg: cervico-vaginal swab Respiratory specimens: sputum, bronchoalveolar lavage (BAL), and endotracheal aspirates

Out of a total of 22,125 clinical specimens, 4603 (22.87%) were bacterial culture positive, and out of 4603 culture-positive isolates, the maximum number of isolates was recovered from pus/wound swabs (1186; 25.8%), followed by urine samples (995; 21.6%) and blood cultures (756; 16.4%). The least number of pathogens were isolated from conjunctival swabs (86; 1.9%). Overall, pus/wound swabs, urine, and blood cultures constituted the predominant sources of pathogenic isolates, together accounting for more than 60% of the total yield. This distribution reflects the common clinical presentations leading to microbiological investigation in our setting (Figure 1).

**Figure 2:**
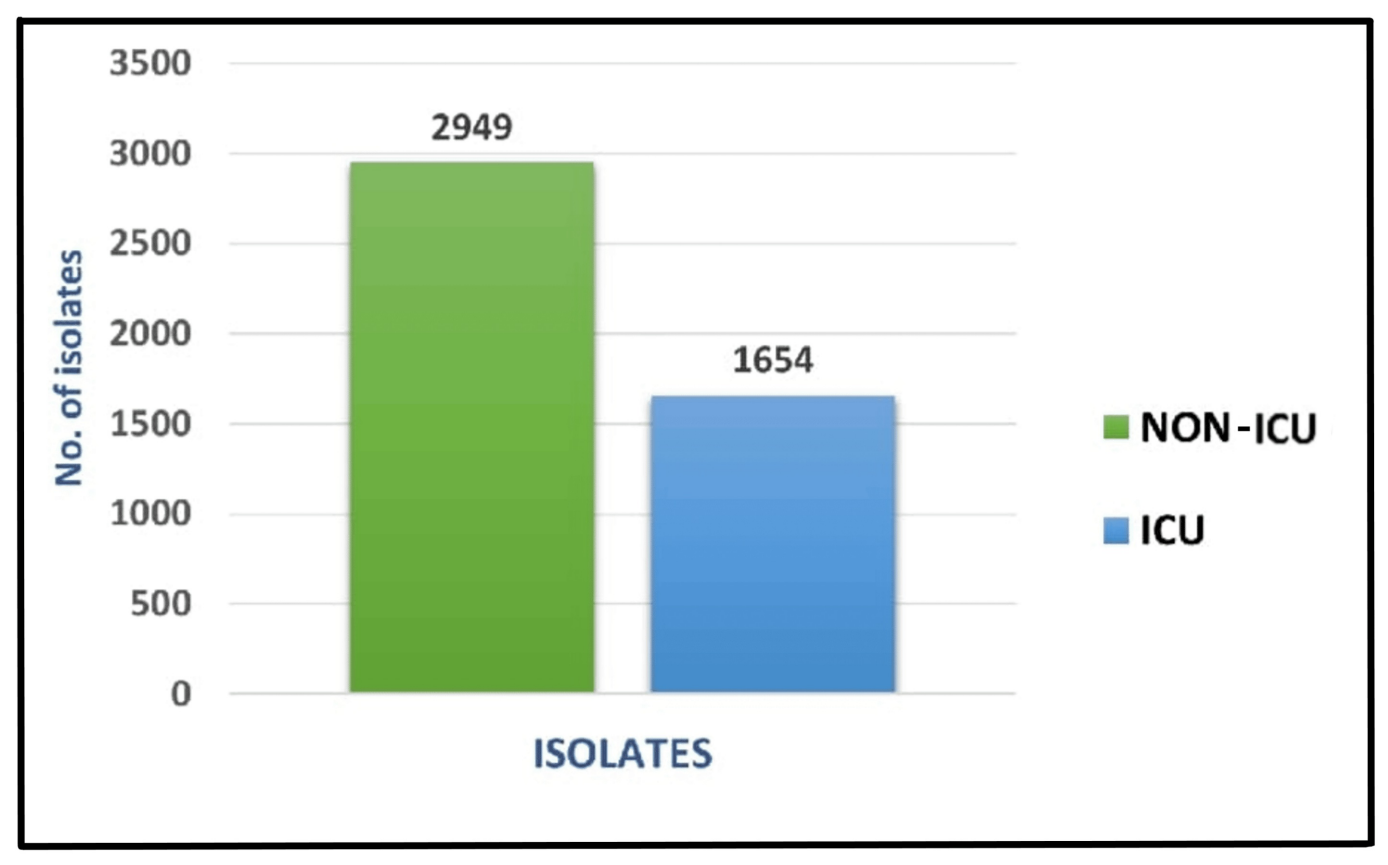
Distribution of isolates among ICU and non-ICU setting (n=4603)

Out of 4603 clinical isolates, 1654 (35.9%) were from ICU settings, while 2949 (64.1%) were recovered from non-ICU areas. Thus, isolates from non-ICU samples predominated over those from ICU samples, although both contributed substantially to the overall distribution (Figure 2).

Out of the total culture-positive isolates, the majority were Gram-negative organisms, accounting for 3702 isolates (80%), while Gram-positive cocci were 901 (20%).

**Figure 3:**
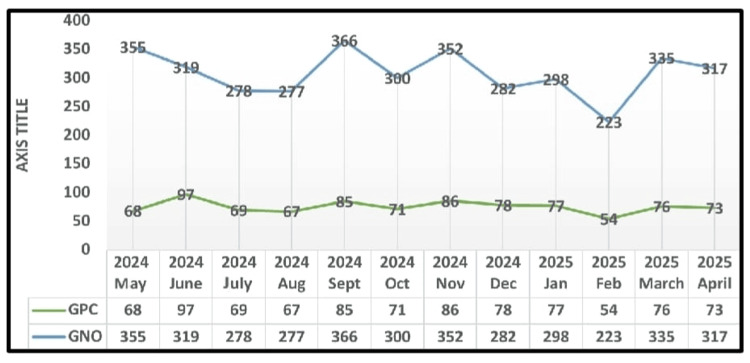
Temporal trends in accordance with GPC and GNO from May 2024 to April 2025 GPC: Gram-positive cocci; GNO: Gram-negative organisms

Between May 2024 and April 2025, the number of Gram-negative organism isolates remained consistently higher than Gram-positive cocci isolates. Gram-negative organism counts peaked in September 2024 (366), followed by a decline to the lowest count in February 2025 (223). Gram-positive cocci counts ranged from 54 to 97, with the highest value in June 2024 (97) and the lowest in February 2025 (54) (Figure 3).

In the study, the most frequently isolated organism was *Escherichia coli* (1321 isolates; 28.7%), followed by *Klebsiella pneumoniae *(1051 isolates; 22.8%) and *Pseudomonas aeruginosa* (785 isolates; 17.05%). Among Gram-positive cocci, *Staphylococcus aureus* accounted for 664 isolates (14.4%), and *Enterococcus *spp. accounted for 152 isolates (3.3%). Month-wise distribution revealed that the maximum number of isolates was reported in September 2024 (451 isolates; 9.8%) and November 2024 (438 isolates; 9.5%), whereas the lowest yield was observed in February 2025 (277 isolates; 6%) (Table 1).

**Table 1:** Distribution of pathogens isolated from May 2024 to April 2025 (n=4603) CoNS: coagulase-negative *Staphylococcus*

Months	May 24 (N=423)	June 24 (N=416)	July 24 (N=347)	Aug 24 (N=344)	Sep 24 (N=451)	Oct 24 (N=371)	Nov 24 (N=438)	Dec 24 (N=360)	Jan 25 (N=374)	Feb 25 (N=278)	Mar 25 (N=411)	Apr 25 (N=390)
Isolates												
*Escherichia coli *(n=1321)	121	125	98	93	134	108	144	103	101	86	110	98
*Klebsiella pneumoniae* (n=1051)	97	87	79	80	96	88	102	84	88	68	97	85
*Enterobacter aerogenes* (n=1)	-	-	-	-	1	-	-	-	-	-	-	-
*Enterobacter cloacae* (n=2)	-	1	-	-	-	-	-	1	-	-	-	-
*Citrobacter *sp. (n=4)	-	2	-	-	-	1	-	-	-	-	1	-
*Serratia *sp. (n=4)	-	1	1	-	1	-	1	-	-	-	-	-
*Proteus mirabilis* (n=21)	-	4	3	3	-	1	2	1	2	-	3	2
*Proteus vulgaris* (n=5)	1	-	1	-	1	-	1	-	-	-	-	1
*Providencia *spp. (n=3)	-	-	-	-	1	-	-	-	1	-	1	-
*Morganella morganii* (n=2)	-	1	-	-	-	-	-	-	-	-	-	1
*Salmonella typhi* (n=11)	-	3	2	2	1	1	-	-	1	-	1	-
*Vibrio cholerae* (n=13)	1	7	3	1	-	-	-	-	-	-	-	1
*Pseudomonas aeruginosa* (n=785)	81	67	57	57	83	67	65	58	66	42	72	70
*Acinetobacter *spp. (n=479)	54	21	34	41	48	34	37	35	38	28	50	59
*Staphylococcus aureus* (n=664)	54	64	48	51	65	54	64	56	61	43	52	52
CoNS (n=85)	4	15	9	8	5	8	8	5	5	7	7	4
*Enterococcus *spp. (n=152)	10	18	12	8	15	9	14	17	11	4	17	17

Among the *Enterobacterales*, *Escherichia coli* (n=1321) and *Klebsiella pneumoniae* (n=1051) showed high resistance to first- and second-generation cephalosporins, with 88-89% resistance to cefazolin and cefuroxime, while resistance to third-generation cephalosporins such as ceftriaxone and cefotaxime ranged from 73% to 76%. Carbapenem resistance was notable, with imipenem resistance observed in 51-52% and meropenem in 44-51% of isolates. Aminoglycosides demonstrated limited activity, with gentamicin and amikacin resistance exceeding 30%. Fluoroquinolone resistance was widespread (44-54% for ciprofloxacin and levofloxacin). Nitrofurantoin showed (16-24%) activity, while fosfomycin (10%) retained activity against *E. coli* only. All isolates remained uniformly susceptible to colistin. Other non-*Enterobacterales* showed high resistance to cephalosporins (89%), followed by aztreonam (68%), and showed the least resistance to nitrofurantoin (21%) (Table 2).

**Table 2:** Antibiotic resistance pattern in lactose-fermenting Gram-negative isolates IR: intrinsic resistance; NT: not tested; MIC: only MIC is recommended for the isolate; NR: not recommended by the Clinical and Laboratory Standards Institute; U: tested only in urinary isolates

Antibiotics	*Escherichia coli* (n=1321) %	*Klebsiella pneumoniae* (n=1051) %	Other *Enterobacterales* (n=43) %
Ampicillin	98	IR	IR
Cefazolin	88	89	IR
Cefuroxime	86	88	89
Cefoxitin	78	80	IR
Ceftriaxone	76	78	76
Cefotaxime	73	76	76
Cefixime	77	77	78
Ceftazidime	72	73	72
Cefepime	56	55	56
Imipenem	52	51	52
Meropenem	44	51	48
Aztreonam	18	65	68
Amoxicillin-clavulanate	88	89	IR
Piperacillin-tazobactam	53	52	52
Ampicillin-sulbactam	58	56	IR
Gentamicin	32	36	33
Amikacin	35	33	32
Tetracycline	33	34	33
Ciprofloxacin	48	44	45
Levofloxacin	54	51	51
Cotrimoxazole	43	41	43
Nitrofurantoin (U)	16	24	21
Fosfomycin (U)	10	NR	NR
Colistin (MIC)	0	0	0

Among non-lactose fermenters, *Pseudomonas aeruginosa* (n=785) and *Acinetobacter *spp. (n=479) displayed substantial multidrug resistance. Piperacillin-tazobactam resistance was 34% in *P. aeruginosa *and 43% in *Acinetobacter *spp. Fluoroquinolone resistance was also pronounced, with ciprofloxacin resistance at 25% in *P. aeruginosa* and 42% in *Acinetobacter *spp. Overall, carbapenem resistance was high, with imipenem and meropenem resistance ranging from 41% to 66%. Importantly, colistin retained activity against all tested isolates (Table 3). 

**Table 3:** Antibiotic resistance pattern in non-lactose-fermenting Gram-negative isolates IR: intrinsic resistance; NT: not tested; MIC: only MIC is recommended for the isolate; NR: not recommended by the Clinical and Laboratory Standards Institute; U: tested only in urinary isolates

Antibiotics	*Pseudomonas aeruginosa* (n=785) %	*Acinetobacter *spp. (n=479) %
Ampicillin	NR	NR
Cefazolin	IR	IR
Cefuroxime	IR	IR
Cefoxitin	IR	IR
Ceftriaxone	IR	78
Cefotaxime	IR	76
Cefixime	IR	IR
Ceftazidime	79	68
Cefepime	28	52
Imipenem	45	66
Meropenem	41	55
Aztreonam	56	IR
Amoxicillin-clavulanate	IR	IR
Piperacillin-tazobactam	34	43
Ampicillin-sulbactam	IR	72
Gentamicin	IR	26
Amikacin	IR	24
Tetracycline	NR	NR
Azithromycin	NR	NR
Pefloxacin	NR	NR
Ciprofloxacin	25	42
Levofloxacin	20	38
Chloramphenicol	NR	NR
Cotrimoxazole	IR	28
Nitrofurantoin (U)	IR	IR
Fosfomycin (U)	IR	IR
Colistin (MIC)	0	0

High-level resistance was observed in *Vibrio cholerae* (n=13) from stool samples across multiple classes, with beta-lactam ampicillin reaching 98% resistance. Other key resistance figures include azithromycin (55%), chloramphenicol (45%), and cotrimoxazole and doxycycline (35%) each.

*Salmonella typhi* (n=11) from intestinal as well as extra-intestinal infections showed resistance to ampicillin (78%), cefotaxime (49%), azithromycin (64%), and levofloxacin (64%), while cotrimoxazole showed 46% resistance.

Out of 901 Gram-positive isolates, *Staphylococcus aureus* (n=664) showed high resistance to penicillin (96%), followed by azithromycin (88%), with moderate resistance to erythromycin (34%), ciprofloxacin (21%), and clindamycin (22%). All isolates were susceptible to vancomycin. Other coagulase-negative staphylococci (n=85) also showed very high resistance to penicillin. Enterococci (n=152) showed 90-98% resistance to penicillin and ampicillin, followed by resistance to chloramphenicol (61-65%). Both enterococcal species remained less resistant to linezolid (8-12%) (Table 4).

**Table 4:** Antimicrobial resistance of Gram-positive isolates (n=901) IR: intrinsic resistance; NT: not tested; MIC: only MIC is recommended for the isolate; NR: not recommended by the Clinical and Laboratory Standards Institute; U: tested only in urinary isolates

Antibiotics	*Staphylococcus aureus* (n=664) %	Other coagulase-negative *Staphylococcus *(n=85) %	*Enterococcus faecalis* (n=98) %	*Enterococcus faecium* (n=54) %
Penicillin	96	96	98	98
Ampicillin	NR	NR	90	92
Cefoxitin	48	22	IR	IR
Clindamycin	22	24	IR	IR
Erythromycin	34	32	46	41
Doxycycline	25	22	42	36
Tetracycline	27	21	47	42
Ciprofloxacin	21	5	32	35
Levofloxacin	23	6	25	32
Gentamicin	12	0	IR	IR
High-level gentamycin	NR	NR	32	26
Cotrimoxazole	19	0	IR	IR
Chloramphenicol	75	23	65	61
Vancomycin	0 (MIC)	0 (MIC)	3	5
Teicoplanin	MIC (NT)	MIC (NT)	36	30
Linezolid	8	7	8	12
Azithromycin	88	80	NR	NR
Nitrofurantoin (U)	18	NR	22	24
Fosfomycin (U)	12	NR	16	NR

Among the Gram-positive isolates, MRSA (48.72%) was the most frequent. Methicillin-resistant coagulase-negative staphylococci were 5.32%, reflecting their role in opportunistic and device-related infections. Inducible clindamycin resistance (21.97%) was noteworthy as it may cause therapeutic failures if unrecognized. Vancomycin-resistant enterococci were 0.77% (Table 5).

**Table 5:** Resistance mechanism of Gram-positive isolates

	Resistance mechanisms in Gram-positive cocci isolates	Percentage (100%)
1	Methicillin-resistant *Staphylococcus aureus* (n=439)	48.72
2	Methicillin-resistant coagulase-negative staphylococci (n=48)	5.32
3	Inducible clindamycin resistance (n=198)	21.97
4	Vancomycin-resistant enterococci (n=7)	0.77

The analysis of resistance patterns among the isolates revealed that the majority of organisms (59%) were MDR. These findings highlight a significant burden of AMR, with a considerable proportion of organisms showing resistance to multiple classes of antimicrobial agents.

## Discussion

This one-year surveillance (May 2024-April 2025) of 22,125 clinical specimens provided important insights into hospital trends of bacterial pathogens and their antimicrobial resistance profiles. Gram-negative bacilli (GNB) constituted the majority of isolates, with *Escherichia coli* (35.7%) and *Klebsiella pneumoniae* (28.4%) being predominant, followed by *Pseudomonas aeruginosa* (21.1%) and *Acinetobacter* spp. (13%). This distribution closely parallels the findings of Bai et al., where *Enterobacterales *and non-fermenters were identified as leading pathogens in both nosocomial and community-acquired infections [11].

In our study, *K. pneumoniae* emerged as the second most dominant organism, showing high rates of multidrug resistance. Similar observations were made by Halder et al., who reported that* K. pneumoniae* is particularly frequent in ICU bloodstream infections and poses a therapeutic challenge due to its MDR phenotype [12]. On a global scale, cephalosporin resistance among *K. pneumoniae* bloodstream isolates is estimated at nearly 50%, driving the increased use of second-line agents and thereby fueling further resistance [13]. In the present study, among *Enterobacterales*, resistance rates exceeded 70% for third-generation cephalosporins and approached ~50% for carbapenems. These findings highlight the urgent need for advanced surveillance of resistance mechanisms, including molecular characterization, and also strengthen the case for long-term strategies such as vaccine development against *E. coli* and *K. pneumoniae* [14].

In India, carbapenem resistance rates for *Acinetobacter baumannii* are frequently reported in the 70-90% range and for *Pseudomonas aeruginosa* around 25-40% in many tertiary care series and national surveillance reports [15]. Globally, a 78% increase in hospital-onset carbapenem-resistant *Acinetobacter* spp. infection was identified between 2019 and 2020 in the Centers for Disease Control and Prevention (CDC) preliminary analysis [16]. 

Among Gram-positive organisms, MRSA accounted for nearly half (48.7%) of the *S. aureus *isolates, highlighting its major clinical significance due to limited treatment options. This burden mirrors findings by Ejaz et al., who documented persistently high MRSA prevalence in tertiary care settings [17]. Importantly, very little vancomycin resistance (0.77%) was detected in *Enterococcus* in our study. However, the potential emergence of reduced glycopeptide susceptibility, such as VISA and hVISA strains, though reported at low frequencies (4.6-2.5%) in India [18], underscores the importance of continuous monitoring and stewardship to preserve the efficacy of last-line therapies.

Linezolid resistance (8-12%) among Gram-positive isolates and the emergence of VRE in 5-6% of *E. faecium* in our dataset represent worrisome developments. Sengupta et al. reported VRE in 6.7% of isolates, while linezolid resistance remained rare and was associated with the G2576T 23S rRNA mutation [19]. In contrast, Smout et al., through a meta-analysis, observed a pooled VRE prevalence in India of 12.4%, with a rising trend from 4.8% during 2000-2010 to 14.1% in 2011-2020 [20]. The present data, therefore, indicate a locally significant but comparatively lower burden, warranting vigilant surveillance to prevent escalation.

The resistance profile highlights the narrowing spectrum of reliable therapeutic options. Colistin retained complete activity against GNB in this study, underscoring its continuing role as a salvage therapy. Colistin remained active against all isolates; however, rising reliance warrants close surveillance for emerging resistance. For Gram-positive organisms, vancomycin remains effective against MRSA, while linezolid continues to provide substantial coverage despite emerging resistance. Kulshrestha et al. documented linezolid resistance at 2.8% [21], whereas our finding of 8-12% suggests a concerning local emergence. These differences likely reflect variations in sample size, institutional antibiotic pressure, and local epidemiology, emphasizing the need for cautious and judicious use of these critical agents.

The high empirical use of WHO-designated "Watch" and "Reserve" antibiotics in Indian hospitals, frequently without microbiological confirmation, remains a major driver of resistance, as highlighted by global and regional reviews [22]. This underscores the necessity of robust antimicrobial stewardship programs that promote culture-guided prescribing, rational drug selection, and optimization of antimicrobial therapy.

Non-fermenters, disproportionately isolated from ICU settings in our study, point to their central role in nosocomial outbreaks and ICU-related infections. Strengthened infection prevention strategies, such as strict hand hygiene, rigorous environmental cleaning, and adherence to device bundles, are critical to curbing their spread.

In our study, 59% of isolates were MDR, and the detection of such high proportions of MDR strains signifies a serious therapeutic challenge in hospital settings.

Basak et al., in a study from a tertiary care hospital in Wardha, reported that out of 1060 bacterial strains, 393 (37.1%) were MDR and 146 (13.8%) were extensively drug-resistant (XDR), while no pandrug-resistant (PDR) strains were identified [23]. Such infections are a major cause of prolonged hospitalization, increased treatment costs, and poor patient outcomes, including higher morbidity and mortality [24].

These findings highlight three key implications. First, they underscore the urgent need for hospital-based antibiograms and regular resistance surveillance to guide empirical therapy. Second, they strengthen the case for antimicrobial stewardship programs that discourage the non-evidence-based use of broad-spectrum agents and promote de-escalation once culture results are available. Third, they emphasize the necessity of rigorous infection prevention measures, particularly in ICU settings, to reduce the spread of these highly resistant organisms. Collectively, these results also call for accelerated research into new antimicrobials, vaccines, and alternative approaches to tackle drug-resistant infections.

Taken together, these findings reaffirm the importance of continuous, comprehensive surveillance systems. Integrating data-driven diagnostics, including rapid molecular detection of resistance determinants such as carbapenemase genes or vancomycin resistance markers, can improve timely therapeutic decisions and patient outcomes. Such integration is crucial for improving clinical outcomes and containment of AMR in tertiary care settings.

Being a single-center investigation, the results do not necessarily reflect the resistance patterns of other hospitals or regions. Among non-fermenters, the available conventional resources cover only *Pseudomonas aeruginosa* and *Acinetobacter* spp. Molecular analysis of resistance genes was not performed, which restricted our ability to link phenotypic resistance with underlying genetic mechanisms. Clinical outcomes such as treatment response, hospital stay, or mortality were not evaluated, limiting the assessment of the direct patient impact. In addition, the study covered a one-year period only; therefore, long-term trends in resistance could not be established. Finally, data on antibiotic usage were not included, which could have provided insights into the drivers of resistance observed.

## Conclusions

This surveillance highlights an alarming burden of AMR, with *Enterobacterales *and non-fermenters showing multidrug resistance and Gram-positive pathogens adding to the challenge through MRSA, VRE, and emerging linezolid resistance. Dependence on last-resort agents like colistin reflects the fragility of current treatment options. The high prevalence of MDR isolates underscores the clinical consequences, such as higher mortality, prolonged hospitalization, and rising costs. Addressing this crisis requires strict antimicrobial stewardship, reinforced infection control, and adoption of rapid diagnostics to guide timely therapy. Looking ahead, preventive strategies such as vaccines and novel therapies, including bacteriophages and monoclonal antibodies, are essential. Combating AMR will ultimately rely on three pillars: preserving current drugs, preventing transmission, and preparing future solutions.

## References

[1] (2022). Global burden of bacterial antimicrobial resistance in 2019: a systematic analysis. Lancet.

[2] Uddin TM, Chakraborty AJ, Khusro A (2021). Antibiotic resistance in microbes: history, mechanisms, therapeutic strategies and future prospects. J Infect Public Health.

[3] (2023). Antimicrobial resistance. https://www.cdc.gov/antimicrobial-resistance/index.html.

[4] Rice LB (2010). Progress and challenges in implementing the research on ESKAPE pathogens. Infect Control Hosp Epidemiol.

[5] (2023). UN, global health agencies sound alarm on drug-resistant infections; new recommendations to reduce 'staggering number' of future deaths. https://news.un.org/en/story/2019/04/1037471.

[6] Harun MG, Anwar MM, Sumon SA (2022). Infection prevention and control in tertiary care hospitals of Bangladesh: results from WHO Infection Prevention and Control Assessment Framework (IPCAF). Antimicrob Resist Infect Control.

[7] Collee JG (1996). Mackie & McCartney Practical Medical Microbiology. Mackie & McCartney practical medical microbiology (14th.

[8] (2024). CLSI M100: Performance Standards for Antimicrobial Susceptibility Testing. https://clsi.org/shop/standards/m100/.

[9] (2018). CLSI M45: Methods for Antimicrobial Dilution and Disk Susceptibility Testing of Infrequently Isolated or Fastidious Bacteria. https://clsi.org/shop/standards/m45/.

[10] Sankar SA, Bhat KS, Anand J (2016). Staphylococcus. Essentials of Medical Microbiology.

[11] Bai HJ, Geng QF, Jin F, Yang YL (2024). Epidemiologic analysis of antimicrobial resistance in hospital departments in China from 2022 to 2023. J Health Popul Nutr.

[12] Halder G, Chaudhuri BN, Veeraraghavan B (2025). Antimicrobial resistance and phylogenetic lineages of KPC-2-producing blood-borne Klebsiella pneumoniae subsp. pneumoniae from Kolkata, India during 2015-2024: emergence of Klebsiella pneumoniae subsp. pneumoniae with blaKPC-2, blaNDM, and blaOXA-48-like triple carbapenemases. Microbiol Spectr.

[13] Chong CE, Pham TM, Carey ME (2024). Conference report of the 2024 Antimicrobial Resistance Meeting. NPJ Antimicrob Resist.

[14] Cabrera A, Mason E, Mullins LP, Sadarangani M (2025). Antimicrobial resistance and vaccines in Enterobacteriaceae including extraintestinal pathogenic Escherichia coli and Klebsiella pneumoniae. NPJ Antimicrob Resist.

[15] (2022). Annual Report Antimicrobial Resistance Surveillance Network. https://www.icmr.gov.in/icmrobject/custom_data/pdf/resource-guidelines/AMRSN_Annual_Report_2022.pdf.

[16] Castanheira M, Mendes RE, Gales AC (2023). Global epidemiology and mechanisms of resistance of Acinetobacter baumannii-calcoaceticus complex. Clin Infect Dis.

[17] Ejaz M, Syed MA, Jackson CR, Sharif M, Faryal R (2023). Epidemiology of Staphylococcus aureus non-susceptible to vancomycin in South Asia. Antibiotics (Basel).

[18] Shariati A, Dadashi M, Moghadam MT, van Belkum A, Yaslianifard S, Darban-Sarokhalil D (2020). Global prevalence and distribution of vancomycin resistant, vancomycin intermediate and heterogeneously vancomycin intermediate Staphylococcus aureus clinical isolates: a systematic review and meta-analysis. Sci Rep.

[19] Sengupta M, Sarkar R, Sarkar S, Sengupta M, Ghosh S, Banerjee P (2023). Vancomycin and linezolid-resistant Enterococcus isolates from a tertiary care center in India. Diagnostics (Basel).

[20] Smout E, Palanisamy N, Valappil SP (2023). Prevalence of vancomycin-resistant enterococci in India between 2000 and 2022: a systematic review and meta-analysis. Antimicrob Resist Infect Control.

[21] Kulshrestha N, Ghatak T, Gupta P (2019). Surveillance of health-care workers for nasal carriage to detect multidrug-resistant Staphylococcus app. in a tertiary care center: an observational study. Med J DY Patil Vidyapeeth.

[22] (2025). Superbugs threaten hospitals as antibiotic resistance rises: PGI study. https://timesofindia.indiatimes.com/city/chandigarh/superbugs-threaten-hospitals-as-antibiotic-resistance-rises-pgi-study/articleshow/123418277.cms.

[23] Basak S, Singh P, Rajurkar M (2016). Multidrug resistant and extensively drug resistant bacteria: a study. J Pathog.

[24] Lautenbach E, Patel JB, Bilker WB, Edelstein PH, Fishman NO (2001). Extended-spectrum β-lactamase-producing Escherichia coli and Klebsiella pneumoniae: risk factors for infection and impact of resistance on outcomes. Clin Infect Dis.

